# Dual Function of Par3 in Tumorigenesis

**DOI:** 10.3389/fonc.2022.915957

**Published:** 2022-07-08

**Authors:** Tao Lv, Jiashun Xu, Hemei Yuan, Jianling Wang, Xinni Jiang

**Affiliations:** ^1^Centre for Yunnan Plateau Biological Resources Protection and Utilization, College of Biological Resource and Food Engineering, Qujing Normal University, Qujing, China; ^2^Yunnan Engineering Research Center of Fruit Wine, Qujing Normal University, Qujing, China; ^3^Key Laboratory of Yunnan Province Universities of Qujing Natural History and Early Vertebrate Evolution, College of Biological Resource and Food Engineering, Qujing Normal University, Qujing, China; ^4^College of Chemistry and Environmental Science, Qujing Normal University, Qujing, China; ^5^School of Biological Sciences and Technology, Chengdu Medical College, Chengdu, China

**Keywords:** cell polarity, Par3, dual function, tumor-promoting, tumor-suppressive

## Abstract

Cell maintenance and the establishment of cell polarity involve complicated interactions among multiple protein complexes as well as the regulation of different signaling pathways. As an important cell polarity protein, Par3 is evolutionarily conserved and involved in tight junction formation as well as tumorigenesis. In this review, we aimed to explore the function of Par3 in tumorigenesis. Research has shown that Par3 exhibits dual functions in human cancers, both tumor-promoting and tumor-suppressive. Here, we focus on the activities of Par3 in different stages and types of tumors, aiming to offer a new perspective on the molecular mechanisms that regulate the functions of Par3 in tumor development. Tumor origin, tumor microenvironment, tumor type, cell density, cell–cell contact, and the synergistic effect of Par3 and other tumor-associated signaling pathways may be important reasons for the dual function of Par3. The important role of Par3 in mammalian tumorigenesis and potential signaling pathways is context dependent.

## Introduction

Cell polarity is a fundamental feature of almost all cells (1, 2). Different types of cells employ polarity to orient their behavior in a variety of different processes, including embryogenesis (3), epithelial morphogenesis (4), neuronal differentiation (5), fibroblast migration (6), neuroepithelial morphogenesis (7), and T-cell activation (8), which are thought to rely on a small number of evolutionarily conserved proteins and pathways. The maintenance of cell polarity involves sophisticated interactions between multiple protein complexes as well as the regulation of different signaling pathways. The spatiotemporal characteristics of these interactions control the location and distribution of various membrane proteins, organelles, and cytoskeletal components in an asymmetric manner. Recently, vast studies have shown that cell polarity is related to directed migration, differentiation, proliferation, vector transportation of molecules between cell layers, and activation of immune cells, while a loss of cell polarity is correlated with the occurrence of malignant tumors (9–13). Partition-Defective 3 (Par3) is a PDZ-domain-containing scaffold protein that is evolutionarily conserved and essential for the establishment of the cell polarity of various cell types, such as inner ear hair cells (14), hepatocytes (15), and epithelial cells (10). In this review, we will summarize the complicated and comprehensive role of Par3 in the occurrence and development of different cancer types.

## The Par3 Gene and Its Protein Products

**Figure 1:**
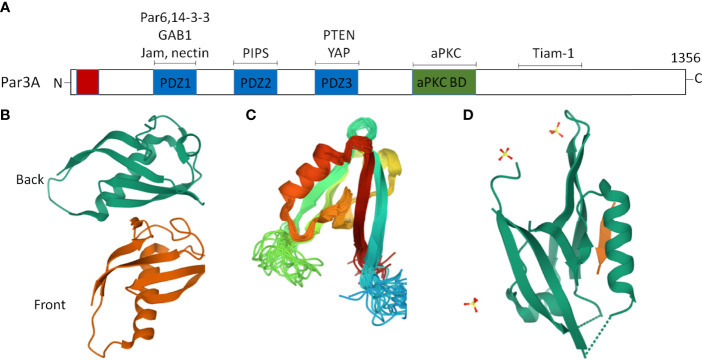
Two dimensional (2D) and partial three-dimensional (3D) structures of Par3. **(A)** Par3 encodes a protein of 1,356 amino acids, consisting of an N-terminal domain, a C-terminal domain, and three PDZ domains, namely, PDZ1, PDZ2, and PDZ3. The interacting proteins of Par3 are indicated above the approximate localization of the binding sites. **(B)** A ribbon diagram of the crystal structure of the two Par-3 NTD molecules in the asymmetric unit (PDB accession number:416P). The two molecules, colored green and brown, respectively, are arranged in a ‘‘front-to-back’’ manner. **(C)** A ribbon diagram of the Par3 PDZ2 domain structure (PDB accession number:2KOM). **(D)** A ribbon representation of the Par3 PDZ3 domain (PDB accession number:6JUE).

The Par proteins were first identified in a *Caenorhabditis elegans* screen for mutants that were defective in the anterior–posterior partitioning of proteins in the early embryo (16). In vertebrates, studies on asymmetric fate determinants, such as Par and Mib (Mindbomb), have gone even further in recent years (17, 18). The Par polarity protein family is composed of seven core members: three kinases [atypical protein kinase C (aPKC), Par1, and Par4], two scaffold proteins (Par3 and Par6), a ring finger protein (Par2), and a 14-3-3 protein (Par5) (19–22). The multidomain scaffolding protein Par3, which contains three PSD-95/Discslarge/ZO-1 (PDZ) domains, an N-terminal dimerization domain (NTD), a C-terminal domain, and an aPKC interaction domain, is an important member of the Par protein family (23, 24). Notably, the crystal structures of the NTD (25), PDZ2 (26), and PDZ3 (27) domains have been successfully resolved (**Figure 1**). The PDZ domains interact with cell-surface proteins such as junctional adhesion molecules (JAMs) (28, 29), Nectin (30), Par6 (31), the adaptor protein GAB1 (32), phosphoinositides (PIPs) (33), the lipid phosphatase PTEN (34), and the Hippo pathway transcription factor YAP (35). The C-terminal domain of Par3 interacts with aPKC and a Rac1 GTPase-specific GTP exchange factor Tiam1 (T lymphoma invasion and metastasis) to inhibit its kinase activity and exchange activity, respectively (23) (**Figure 1**). It is worth noting that the scaffold protein Par3 consists of Par3A (mainly including 180K, 150K, and 100K subtypes) and Par3B. Par3A can interact with Par6 and aPKC, but Par3B cannot (22).

The Par protein family plays an important role in establishing cell polarity and tight junction (TJ) formation in different tissue types and is evolutionarily conserved (36). In *C. elegans*, Par3 and Par6 mediate the formation and maturation of junctions in embryonic morphogenesis (37), while the aPKC complex and Par1 are essential for the polarity of zygotes. In *Drosophila* and mammalian cells, the aPKC complex and Par1 are in different locations among various cell types. The aPKC complex and Par1 localize at the anterior and posterior cortices of *Drosophila* eggs (38, 39), and the apical and basolateral membranes of *Drosophila* epithelial cells (40, 41) and mammalian epithelial cells (42, 43), respectively. Significantly, conserved serine residues on Par3 are phosphorylated by Par1 in *Drosophila* oocytes and follicle cells, resulting in the destabilization of the aPKC complex, and preventing the invasion of the aPKC complex into the posterior and basolateral membranes (38). In mammalian epithelial cells, however, a conserved threonine residue on Par1B is phosphorylated by aPKC, which induces its dissociation from the basolateral membrane (44). Interestingly, in the *C. elegans* embryo, the phosphorylation of Par1 and Par2 by aPKC may also play an important role in the anterior cortex (45). In the transparent zebrafish embryo, Par3 and aPKC are required to promote neurogenic divisions, revealing the essential role of Par3 in the vertebrate neural tube (17). In addition, the proportion of early-born neurons was found to be increased in the absence of Par3 function in the mouse cortex, indicating that Par3 is required to maintain apical progenitors in a proliferative state (46, 47).

## Par3 and Cell Polarity

In the process of morphogenesis, cells undergo a profound reorganization of the cytoskeleton, organelles, cell membrane, and other cell components to form an internal asymmetric axis. In mammalian epithelial cells, the ternary complex consists of three proteins, Par3, Par6, and aPKC, which are located at the top of cells and play an important role in the tight connection of epithelial cells as well as the establishment of cell polarity (22, 48). Thus, this Par complex is a modulator of TJ homeostasis and apical–basal polarity (49). As a key component of the Par complex, Par3 is required for the spatial organization of several important signaling proteins (22).

The adaptor protein Par6 can form a fundamental complex with aPKC, which is delivered to the apical surface by the binding of Par3 to Par6 (50, 51). Furthermore, aPKC can directly interact with Par3, which is essential for the apical localization of aPKC and epithelial organization (51, 52). Activated aPKC triggers JAK or Src kinase to phosphorylate Stat3, which, in turn, induces the expression of MMP, leading to the degradation of ECM and the migration of primary tumors (53). Studies have shown that constitutive activation of aPKC can occur with a loss of Par3 (53–55) as well as nonspecific inhibitors of aPKC (56–58). Notably, the anticancer function of Par3 is partially due to the restriction of aPKC activity (59–61).

Par3 binds to Par6, recruits Par6-associated proteins, and then interacts with Tiam1 to form TJs, providing an anchorage for the apical–basolateral border assembly of the Par complex (62). The localization of Tiam1 regulated by Par3 at the TJs is necessary for maintaining cell polarity. In epithelial cells, the destruction of TJs leads to the loss of cell polarity. Par3 can cross-talk with Rho GTPase signaling through interaction with the Tiam/Rac signaling pathway. In keratinocytes, Tiam1 and Rac collaborate with the Par complex to regulate TJ biogenesis and persistent migration (63).

Proteins of the Par complex are located in the original TJ, regulating their maturation and localization concerning basolateral and apical membrane domains in epithelial cells as well as the maintenance of apical–basal polarity (64–67). RNA interference with Par3 expression leads to a dramatic destruction of TJs in mammalian epithelial cells (62). The destruction of TJs during ATP depletion results in a decrease in Par3 phosphorylation and Par complex dysfunction in MDCK cells (68). Taken together, these findings show that a mutation or loss of Par complex proteins is crucial in apical–basal polarity formation, resulting in defects in establishing apical identity.

## Dual Function of Par3 Protein

In various epithelial cells, expression changes in any Par complex gene can lead to a disruption of apical–basal polarity (69). Among the three major complexes [i.e., the Scribble, Par, and Crumbs polarity complexes, which are involved in regulating the apical–basal polarity of epithelial cells (12)], the Par complex is implicated in tumorigenesis (70, 71). Recently, increasing evidence has suggested that Par3 also exerts complex-independent functions. In an early study in the *Drosophila* ovary, a loss of Par3 resulted in border cell cluster disorganization and impaired migration (72). According to other research, a loss of Par3 can also reduce tumor functions (73). It is a remarkable fact that a loss of polarity is considered a prerequisite for tumor formation and progression. A loss of Par3 can promote tumor metastasis in breast cancer (23). In summary, Par3 serves as a tumor promoter in radiation-induced retinal carcinoma (74) and ovarian cancer (75) and a tumor suppressor in most other cancer types, including breast cancer (23, 76, 77), thyroid cancer (78), lung cancer (79–81), glioma (82), esophageal cancer (83, 84), endometrioid endometrial carcinoma (85), cervical cancer (86), and pancreatic cancer (87). Interestingly, research has also shown that Par3 has dual functions in skin cancer (88–90) and prostate cancer (73, 91), with both pro-oncogenic and tumor-suppressive functions (90). These findings hint that Par3 may play a dual role in tumorigenesis (89, 92). Furthermore, Par3 is mutated or overexpressed in several human cancers (84, 93). In squamous carcinomas and glioblastomas, the Par3 gene is mutated both in cell lines and in primary tumors (93). In addition, a homozygous deletion in Par3 was found at the chromosomal region 10p11 in human esophageal squamous cell carcinoma (84). In lung squamous cell carcinoma, tumor-specific Par3 mutations were revealed in both patient samples and cell lines (81). The amplification of the Par3 gene was also observed in radiation-induced retinal carcinoma (74).

Except in some tumor types, Par3 expression also varies at different stages of tumors (92). The expression levels of Par3 were examined in 25 normal brain tissues and 43 glioblastoma tissues from the TCGA dataset and a significantly reduced expression of Par3 was observed in tumor tissues (82). Notably, this reduction in Par3 expression was intensified in higher-grade tumors, thus making Par3 a predictor of survival rates in this cohort. Coincidentally, a reduction in Par3 expression was also observed in lung adenocarcinoma compared to normal tissue, leading to lymph node metastasis and poor disease-free survival (80). In contrast, an upregulation of Par3 levels was observed in the advanced stage of ovarian cancer (75).

## Par3 Promotes the Development of Tumors

**Figure 2:**
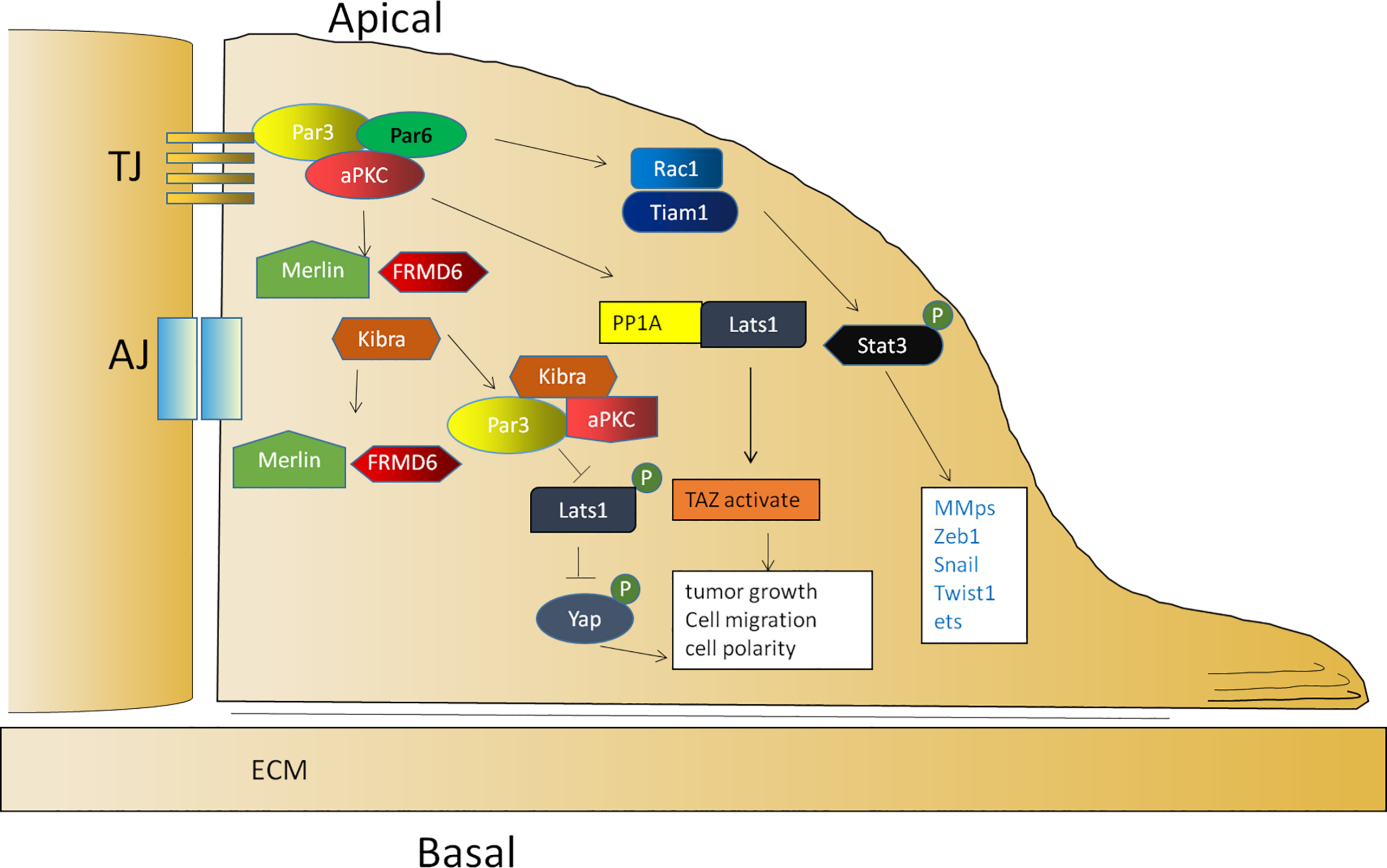
The function of Par3 in tumor promotion. The activation of Rac1 is mediated by Par3/aPKCλ, which is necessary to promote the activation of Stat3. Par3 interacts with PP1A and the Hippo pathway kinase Lats1 to induce its dephosphorylation and thereby lead to the activation of TAZ in the cytoplasm. Par3 also sequesters Kibra to form a Par3/aPKC/Kibra complex, leading to the dissociation of the canonical Kibra/Merlin/FRMD6 complex and a decrease in the phosphorylation of Lats, resulting in the dephosphorylation of Yap for cytoplasmic arrest.

The Par complex is required for neuroblast and epithelial polarization during *Drosophila* embryogenesis and regulates various modes of polarization during neuronal development, migration, and TJ formation in vertebrates. According to early studies, a loss of Par3 weakens the migration ability of the *Drosophila* ovary (72). Soon afterward, researchers also showed that Par3 expression was significantly upregulated in clinical metastatic prostate cancer (73), hepatocellular carcinoma (94), ovarian cancer (75), colorectal cancer (95), and clear cell renal cell carcinoma (96), which was associated with a poor prognosis (94). In a recent study of skin carcinogenesis, Par3 and aPKCλ functioned as a complex to promote tumorigenesis. During tumor initiation, Par3/aPKCλ synergistically promoted Akt, ERK, and NF-κB signal transduction to maintain cell growth (89). Similarly, the ERK- and Akt-mediated growth and survival signals promoted by Par3 can counterbalance apoptotic signaling in skin tumorigenesis (90). In the stage of inflammatory tumor formation, Par3/aPKCλ also synergistically promoted Stat3 activation and accelerated proliferation (89). In addition, Par3 is also involved in the activation of Rac1 (21), which is necessary to promote the activation of Stat3 (97). These observations demonstrate the importance of Par3 in tumorigenesis (**Figure 2**).

Par3 is required for tumor growth according to vast evidence. Par3 promotes tumor growth through the interaction between PP1A and the Hippo pathway kinase Lats1 to induce Lats1 dephosphorylation and inactivation in response to cell contact and cell polarity signals, thereby leading to the dephosphorylation and activation of TAZ in the cytoplasm (98). In addition, Par3 can sequester Kibra to form a noncanonical Par3/aPKC/Kibra complex, resulting in the dissociation of the canonical Kibra/Merlin/FRMD6 complex and a decrease in the phosphorylation of Lats to promote tumor metastasis (73). In prostate cancer, the dissociation of the Par3/aPKC/Kibra complex caused by the downregulation of Par3 activates the Hippo pathway by restoring the phosphorylation of Lats and then leads to the phosphorylation of Yap for a cytoplasmic arrest (73) (**Figure 2**). In another study, Par3 was significantly upregulated in KSHV-infected primary B cells. The knockdown of Par3 led to reduced cell proliferation and increased apoptotic induction. The level of Snail was elevated, while the level of E-cadherin was reduced in the presence of the latency-associated nuclear antigen (LANA) or Par3. The knockdown of Snail simultaneously resulted in reduced expression of LANA and Par3 as well as enhanced expression of E-cadherin simultaneously (99). Collectively, the findings of these studies indicated that maintaining an appropriate expression level of Par3 is crucial for promoting tumor initiation and progression.

## Par3 in Tumor Suppression

In early studies of *Drosophila*, polarity proteins were considered tumor suppressors (100). Mutant polarity proteins or a loss of polarity genes, as well as cooperation between polarity and carcinogenic proteins, such as oncogenic Ras^V12^ (90, 101–103) and ErbB2 (23), results in aggressive and metastatic tumors. Subsequently, Par3 has been reported as a mammalian tumor suppressor (23, 90). Par3 is reduced or lost in a variety of cancer tissues including cervical cancer (86), lung adenocarcinoma (80), thyroid tumor (78), and human breast cancer tissues (77). In addition, the overexpression of Par3 results in the inhibition of the proliferation of esophageal cancer cells and intrauterine membrane carcinoma cells (83, 85) as well as the promotion of tumor cell apoptosis (83). Furthermore, a loss of Par3 leads to active proliferation in many tumor cells (23, 75, 78, 80, 85, 88). Par3-deficient mice are prone to increased rates of keratoacanthoma formation (90).

**Figure 3:**
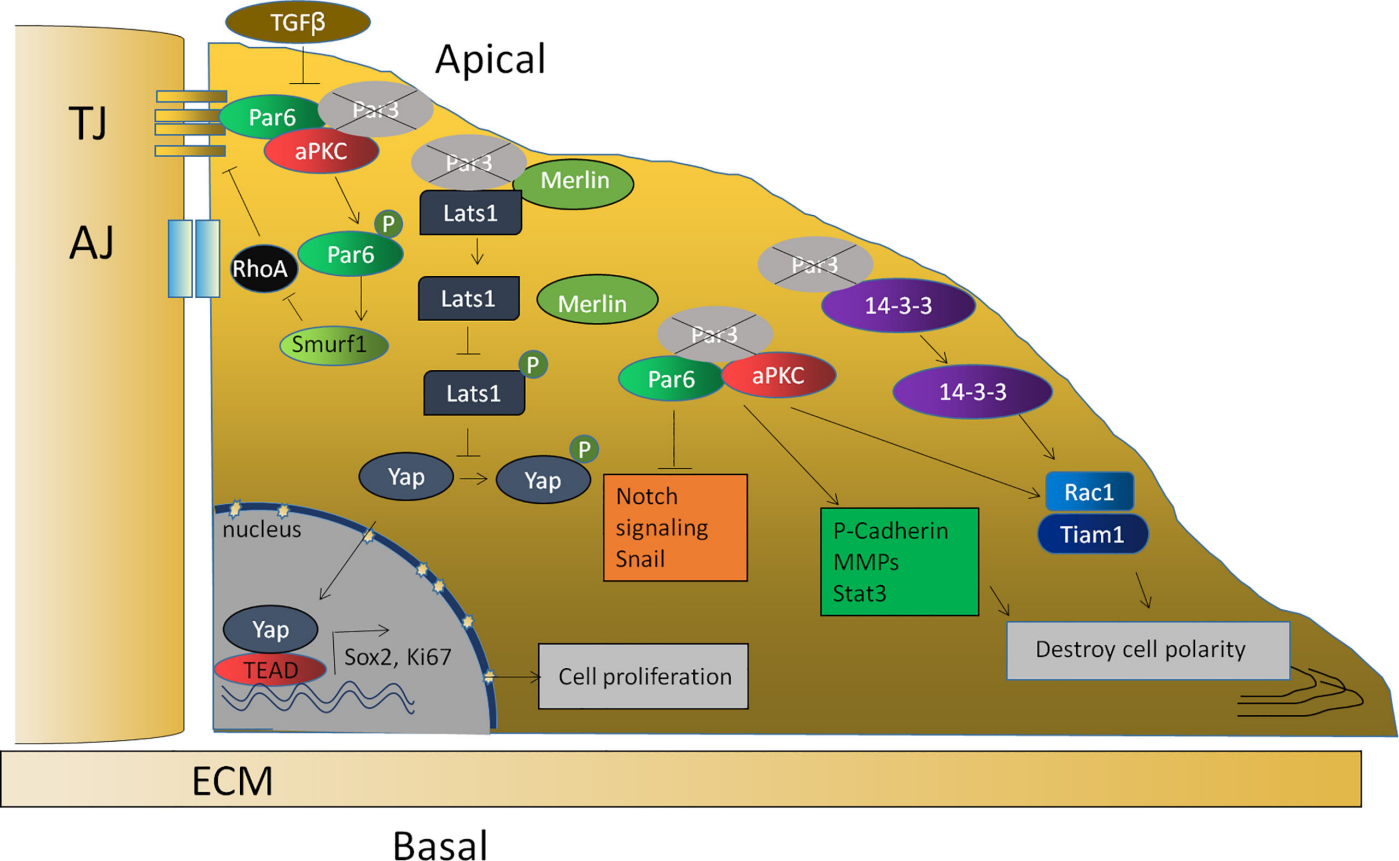
The function of Par3 in tumor suppression. The loss of Par3 in tumor cells is related to the abnormal expression of some important genes, such as P-cadherin, Snail1, and MMP9, and the abnormal regulation of some signaling pathways, such as the Stat3, Tiam1/Rac1, and Notch signaling pathways. The loss of Par3 also leads to the dissociation of the Par3/Merlin/Lats1 complex, leading to decreased Hippo pathway signaling and enhanced nuclear translocation of Yap, resulting in the transcriptional activation of Ki-67 and Sox2. The phosphorylation of Par-6, which is regulated by TGF-β1, can recruit the ubiquitin ligase Smurf1 to the receptor and lead to the localized degradation of RhoA GTPase, thereby leading to the disruption of the tight junction.

To clarify the mechanism, enormous evidence has also shown that the proliferation induced by a loss of Par3 in tumor cells is related to the abnormal expression of some important genes and the abnormal regulation of some signaling pathways, including an upregulation of P-cadherin (88) and MMP9 (77), downregulation of SNAIL1 (104), an activation of Stat3 (77) and the Tiam1/Rac1 signaling pathway (76), and a decrease in the Notch signaling pathway (85). Furthermore, the Par complex is also closely related to EMT in anaplastic thyroid cancer cells and breast cancer cells (78, 105). Furthermore, the binding of Par3 to 14-3-3ζ protein prevented Tiam1, which is responsible for Rac1 activation, from binding to 14-3-3ζ. Therefore, the knockdown of 14-3-3ζ inhibits Tiam1/Rac-GTP activation and blocks the invasive behavior of cells lacking Par3 (80). In addition, a loss of Par3 leads to the dissociation of the Par3/Merlin/Lats1 complex, consequently inhibiting the phosphorylation of Lats1 to attenuate the Hippo pathway and enhancing nuclear translocation of Yes-associated protein (YAP), which promotes cell proliferation and symmetrical cell divisions through transcriptional activation of Ki-67 and Sox2 (91) (**Figure 3**).

According to related reports, TGF-β suppresses the gene expression of E-cadherin, ZO-1, claudin, occludin, and Par3 (66). The decreased Par3 expression subsequently results in the redistribution of the Par-6–aPKC complex from the cell membrane to the cytoplasm. The downregulation of Par-3 and the subsequent disruption of Par complex integrity might be one mechanism by which TGF-β1 destroys cell polarity and cell junctions during EMT (66). The lack of these transmembrane proteins would thus result in the disassembly of cell–cell adhesions. Previous studies have also reported that TGF-β1 can regulate the phosphorylation of Par-6. Phosphorylated Par-6 can recruit the ubiquitin ligase Smurf1 (Smad ubiquitin regulatory factor 1) to the receptor and lead to localized degradation of RhoA GTPase, which is required for the disruption of TJs during EMT (106). Since Par3 interacts with Par-6, the downregulation of Par-3 induced by TGF-β releases Par-6 and thus allows it to be phosphorylated by TGF-β (**Figure 3**). Recent evidence shows that apical–basal polarity, which inhibits EMT and tumor metastasis through Par complex-mediated Snail degradation, functions as a critical checkpoint of EMT to precisely control epithelial–mesenchymal plasticity during tumor metastasis (104). These investigations indicated that Par3 exhibits tumor-suppressive activities and loss of Par3 may contribute to tumorigenesis.

## Concluding Remarks: A Double-Dealer Depending on Context

**Figure 4:**
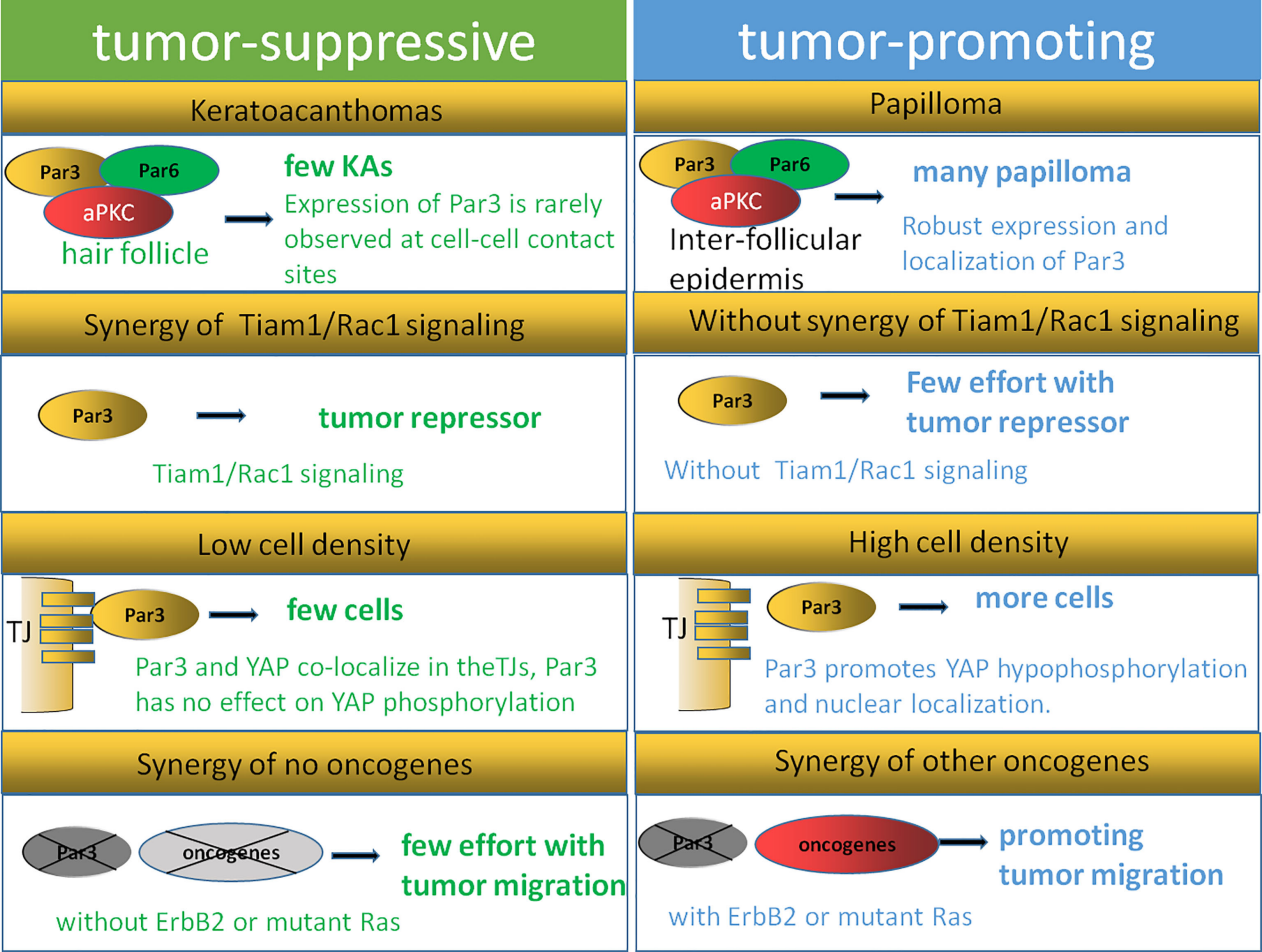
Dual function model of Par3. The expression of Par3 is rarely observed at cell–cell contact sites during keratoacanthoma formation, while robust expression and localization of Par3 are strongly correlated with papilloma formation. Tiam1/Rac signaling can regulate the Par3 complex in keratinocytes, while the inhibitory function of Par3 on keratoacanthoma formation is not observed in Tiam1- and Rac1-deficient mice, indicating that Tiam/Rac signaling is crucial for Par3 function as a tumor repressor. A Par3–YAP complex is reported to regulate the Hippo–YAP pathway in a manner dependent on cell density and cell–cell contact. The dual function of Par3 is related to its synergistic effects with other oncogenes, such as Ras and ErbB2.

In human tumors, the expression of polarity proteins is frequently altered, although this seems to be highly context dependent. Scribble complex proteins often show their tumor-suppressive function in invertebrates (107, 108). aPKC seems to be required for the transformation and tumorigenesis of cancer cells due to its pro-oncogenic functions (109). Par-6 activates aPKC by coupling its localization and activation to precisely control cell polarity (110). Concerning the Par3 protein, its expression is related to both tumor promotion and tumor inhibition under different conditions. The high expression of Par3 protein in a variety of tumors reveals its function of promoting carcinogenesis (75, 95, 111, 112), and is considered to be particularly important because it is necessary for the transformation and tumorigenesis of many cancer cells (89, 90, 95, 98, 99). However, Par3 expression is also frequently downregulated in the primary tumors of various carcinoma types. As reported, Par3 shows the function of higher-grade tumor inhibition with the activation of the ErbB2 or Ras pathway (77, 90). A loss of Par3 results in the dissociation of the Par complex as well as the loss of epithelial polarity (23, 77). Four reasons can be summarized to explain the phenomenon that Par3 exhibits both tumor-promoting and tumor-suppressing actions according to existing research (**Figure 4**).

First, cellular origin and microenvironments affect the dual function of Par3 in tumorigenesis. As reported, papillomas arise mostly from the suprabasal layers of the interfollicular epidermis, while keratoacanthomas originate from the hair follicle (113). A reduced rate of papilloma formation with impaired proliferation and survival signaling is observed in Par3-deficient mice, while an increased rate of keratoacanthoma formation is also observed in Par3-knockout mice, suggesting a dual function of Par3 (90). Par3 is expressed in both the interfollicular epidermis and hair follicles but may act at different intracellular sites to affect papilloma and keratoacanthoma formation, respectively. As reported, the expression of Par3 is rarely observed at cell–cell contact sites during keratoacanthoma formation, while robust expression and localization of Par3 are strongly correlated with the formation of papillomas. Tumor outcomes can be defined by the various microenvironments the cancer cells face. In the basal layers of Par3-knockout epidermal tissues as well as Par3-deficient keratoacanthomas, abnormally high levels of phosphorylated active CRaf were observed in a distinct vesicular pattern, which was moderately detected at cell–cell contacts of the suprabasal layers in wild-type epidermal tissue and only very weakly present in wild-type papilloma tissue (90). The findings of the above studies suggest that the opposite functions of Par3 in tumorigenesis depend on tumor origins and microenvironments.

In addition, the dual functions performed by Par3 may also be affected by the Tiam1/Rac1 pathway. A recent study showed that a loss of Par3 inhibited the formation and growth of papillomas and promoted tumor cell apoptosis in the process of skin tumorigenesis in mice. In contrast, Par3-deficient mice were predisposed to the formation of keratoacanthomas (90). Similarly, the knockdown of the cell polarity protein Tiam1 in epithelial MDCK cells contributed to a loss of apical–basal cell polarity as well as EMT (114). The tumors of Tiam1-deficient mice were highly invasive, effectively linking the loss of Tiam1 expression to EMT *in vivo* (115). However, a loss of Tiam1 is not always associated with tumor progression. As reported by other researchers, increased Tiam1 protein levels were also correlated with the invasive and metastatic growth of some human breast and colon tumors (116, 117). These contradictory findings might be explained by the aberrant localization of Tiam1 away from cell–cell adhesions, for instance, because of the depletion of Par3. According to reports, Tiam1/Rac signaling could regulate the Par3 complex in keratinocytes, while the inhibitory function of Par3 on keratoacanthoma formation was not observed in Tiam1- and Rac1-deficient mice (118), indicating that Tiam/Rac signaling is crucial for Par3 function as a tumor repressor. This leaves the possibility that the dual function of Par3 could be influenced by Tiam1/Rac1 depending on tumor type.

Moreover, cell density and cell–cell contact could also help to explain the dual function of Par3. Recent findings have illustrated the crucial role of the Hippo–YAP pathway in proliferation mediated by cell–cell contact in cancer cells as well as normally developing tissues (119–121). A Par3–YAP complex has been reported to regulate the Hippo–YAP pathway in a manner dependent on cell density and cell–cell contact. As the dynamic subcellular colocalization of Par3 and YAP was regulated by cell density, Par3 activated YAP signaling to mediate cell proliferation at low cell density but not at high cell density. This Par3–YAP complex could recruit PP1A and LATS1/2 to promote YAP hypophosphorylation and nuclear localization. The dual function of Par3 in regulating YAP phosphorylation and activation may also explain the dual function of Par3 in tumorigenesis (35).

Finally, the dual function of Par3 is related to the synergy of other oncogenes, such as Ras and ErbB2. In the DMBA/TPA tumor mouse model involving Ras mutations, Par3 could serve either as a tumor promoter in papilloma formation or as a tumor suppressor in keratoacanthomas formation (90). As introduced in the article, Par3 deficiency resulted in reduced papilloma formation and growth in a Ras-mediated mouse model. Par3 mediated its tumor-promoting activity through the regulation of growth and survival since Par3 deletion increased apoptosis and reduces growth *in vivo* and *in vitro*. This finding is consistent with a study in *D. melanogaster*, which showed that Ras mutation combined with mutations in genes of the Scribble complex caused a loss of apical–basal polarity and neoplastic outgrowth (70). Evidence has also shown that the knockdown of Par3 in conjunction with oncogenic GFP-tagged Ras^61L^ significantly reduced tumor latency compared to GFP-Ras^61L^ alone, indicating that Par3 promoted tumorigenesis by cooperating with oncogenic H-Ras in breast tumorigenesis (77). Furthermore, Par3-deficient mice did not develop spontaneous skin tumors, indicating that Par3 dysfunction alone in mice was not sufficient to drive tumorigenesis (90). Thus, Par3 is likely to promote Ras-induced cell growth and apoptotic resistance, giving rise to a tumor, indicating that the dual function of Par3 may depend on whether the Ras pathway is activated. In addition, by cooperating with ErbB2, a loss of Par3 can inhibit the junction stability of E-cadherin and disrupt cell–cell junctions and cell–cell cohesion through the Tiam1/Rac-GTP pathway, resulting in accelerated metastasis of breast cancer *in vivo*. These findings indicate that a loss of Par3 promotes the metastatic behavior of ErbB2-induced tumor epithelial cells by decreasing cell–cell cohesion (23).

In conclusion, the important role of Par3 in mammalian tumorigenesis and potential signaling pathways is context dependent. Par3 exhibits both pro-oncogenic and tumor-suppressive actions in different stages and types of cancer. Par3 dysfunction may differentially affect tumor outcomes in different tissues depending on tumor origin, tumor microenvironment, tumor type, cell density, cell–cell contact, and the synergy of other tumor-associated signaling pathways.

## Author Contributions

TL, HY, JW, and XJ collected the related paper and drafted the manuscript. JX prepared **Figures 1**–**4**, and TL and XJ participated in the design of the review and draft of the manuscript. All authors read and approved the final manuscript.

## Funding

This work was supported by the National Natural Science Foundation of China (NSFC) (grants 31801175 and 81601602), Yunnan Fundamental Research Projects (202101AT070043), Fund of Chengdu Medical College (CYZ16-03), and Key Project of the Education Department of Sichuan Province (18ZA0162).

## Conflict of Interest

The authors declare that the research was conducted in the absence of any commercial or financial relationships that could be construed as a potential conflict of interest.

## Publisher’s Note

All claims expressed in this article are solely those of the authors and do not necessarily represent those of their affiliated organizations, or those of the publisher, the editors and the reviewers. Any product that may be evaluated in this article, or claim that may be made by its manufacturer, is not guaranteed or endorsed by the publisher.
